# Supplementary material for: Inferring gene regulatory networks using transcriptional profiles as dynamical attractors
Source: PLoS Comput Biol. 2023 Aug 22;19(8):e1010991. doi: 10.1371/journal.pcbi.1010991 (PMC10473541; doi:10.1371/journal.pcbi.1010991)
Supplement: S1 Text — Fig A. Positive correlation between Anet similarity (Hamming distance on the horizontal axis) and attractor profiles similarity (attractor distance on the vertical axis). Each column in the box plots (A-E) contains 1000 random Anetmut mutated from the 5 Anetref consisting of 5–9 genes. Fig B. Demonstration of multiple-TF regulation. The x-axis and y-axis are the protein concentration of two activators or repressors, and the z-axis shows the outcome of the regulation function. (A) Two independent activators. (B) Two independent repressors. (C) Two synergistic activators. (D) Two synergistic repressors. Fig C. Sensitivity tests for the kinetic parameters, (A) rates of transcription, (B) mRNA degradation, (C) translation, and (D) protein degradation. Each of these parameters was perturbed by 50% of their original values and used to generate the correlation between Anet similarity (Hamming distance on the horizontal axis) and attractor profiles similarity (attractor distance on the vertical axis). Fig D. Five GRN architectures were arbitrarily generated as references in the in silico test. They have five-nine (A-E) genes and no self-regulatory edges. The pointed arrows represent activating and the blunt arrows represent repressing regulatory interactions. Fig E. The non-autoregulation in silico test comparison results in F1 score (upper panel), AUROC (middle panel), and AUPRC (bottom panel). The F1 scores are calculated using a threshold cutoff of 0.5 for all models. Best performances are marked by asterisks for symmetric and asymmetric methods. Fig F. Accuracy distributions of the fully trained and directed GRNs determined by the ChIP data in C. albicans. Each distribution contains 30 GRN samples. The fully trained GRNs were solely inferred by the transcriptional profiles while the directed GRNs were also constrained by the ChIP data. Performing equally well on reproducing the transcriptional profiles, the direct GRNs showed a significant increase compared to the fully [file pcbi.1010991.s001.pdf]

Inferring gene regulatory networks using transcriptional profiles as dynamical attractors

Ruihao Li[1], Jordan C. Rozum[2], Morgan M. Quail[1], Mohammad N. Qasim[1], Suzanne S. Sindi[3], Clarissa J. Nobile[4,7], Réka Albert[5,6,*] and Aaron D. Hernday[4,7,*]

[1]Quantitative and Systems Biology Graduate Program, University of California, Merced, Merced, California, United States of America
[2]Department of Systems Science and Industrial Engineering, Binghamton University (State University of New York), Binghamton, New York, United States of America
[3]Department of Applied Mathematics, University of California, Merced, Merced, California, United States of America
[4]Department of Molecular Cell Biology, University of California, Merced, Merced, California, United States of America
[5]Department of Physics, Pennsylvania State University, University Park, University Park, Pennsylvania, United States of America
[6]Department of Biology, Pennsylvania State University, University Park, University Park, Pennsylvania, United States of America
[7]Health Sciences Research Institute, University of California, Merced, Merced, California, United States of America
[*]E-mail: rza1@psu.edu (RA) and ahernday@ucmerced.edu (ADH)

Additional details of parameter estimation and methods used in this study

While the A_{net} and f_0 are iteratively trained by the evolutionary algorithm, the parameters V_{min}, T, and k are directly estimated/calculated by the experimentally derived data, which are the input transcription profiles, V_{max}, translation, and degradation rates. The derivation process was initially removed due to length limits of the manuscript, however this information has now been included in the supplement.

Due to the fact that the A_{net} and the values of some system parameters are often unknown in practice, we are going to make use of the measurable kinetic parameters (including V_{max}, V_{trl}, D_{mRNA}, and $D_{protein}$) and the steady-state transcription profiles to estimate the unknown parameters, which are V_{min}, T, f_0, and k. Since the transcription profiles are assumed to be steady states, the derivatives for all mRNAs and proteins are zero. First, the V_{min} of a gene is estimated by the minimal expression level of the gene across all the samples, as shown in Eq A below.

$$V_{i,min} - \min(I_{i,*}) \cdot D_{i,mRNA} = 0. \tag{A}$$

Therefore, given the $I_{n\times m}$ and D_{mRNA}, we can calculate the V_{min} using Eq B:

$$V_{i,min} = \min(I_{i,*}) \cdot D_{i,mRNA}. \tag{B}$$

Second, without other prior knowledge, we have to assume that when the genes are under TF regulation, they have the same chance to be activated or inhibited. Hence, the T of the TFs is calculated by their average expression levels using the differential equation for protein (Eq 2 in the manuscript), which leads to Eq C:

$$T_{*,i} = \frac{\frac{1}{2} \cdot (\max(I_{i,*}) + \min(I_{i,*})) \cdot V_{i,trl}}{D_{i,protein}}. \tag{C}$$

Third, we assume that the number of input transcription profiles is sufficient and the expression levels of fully activated, or inhibited genes were included in the observed data. When $[P] = [P]_{max}$, the outcome of Eq D, denoted by q, should be close to 1. Therefore, we can calculate the Hill coefficient using Eq E.

$$\frac{[P]^k}{[P]^k + T^k} = q. \tag{D}$$

$$k = \frac{\log \frac{1-q}{q}}{\log \frac{T_i}{[P]}}. \tag{E}$$

$[P]_{max}$ can be calculated by the maximal expression level, translation and protein degradation rates based on the steady-state assumption:

$$[P]_{max} = \frac{V_{trl} \cdot \max(I_{i,*})}{D_{protein}}. \tag{F}$$

We have estimated the V_{min}, T, and k by $I_{n\times m}$ and other known parameters. The last unknown parameter, f_0, is inferred along with the A_{net}. Under the steady-state assumption, the derivative $\frac{d[R]}{dt}$ in Eq 1 equals 0. We can rewrite Eq 1 to calculate the f_0:

$$f_0 = \frac{\frac{D_{i,mRNA} \cdot I_{i,j} - V_{i,min}}{V_{i,max} - V_{i,min}} - C_A \cdot C_R}{C_A + C_R - 2 \cdot C_A \cdot C_R}. \tag{G}$$

We calculated the variance of the outcome networks in 30 inferences using the equation:

$$Variance_{AM} = \frac{\sum_{j=1}^{numGene^2} \mathrm{Var}\left(AM_{*,j}\right)}{numGene^2}, \tag{H}$$

where the $numGene$ is the number of genes in the network and the $\mathrm{Var}\left(AM_{*,j}\right)$ represents the variance of the jth elements in all 30 adjacency matrices. The result is given in Table G. Since the maximum possible variance for a trinary string is 1, which occurs when there are equal numbers of 1s and -1s, and no 0s, and an expected variance of $\frac{2}{3}$ (for equal numbers of 1s, -1s, and 0s), a variance of approximately 0.3 can be considered relatively low, especially considering that the problem is highly under-constrained. Also, we can observe in Fig G that a majority of the elements in the adjacency matrix have a variance between 0.2 to 0.4. Some are highly conserved while some are very flexible, suggesting that the conserved edges play an important role in generating the attractors.

$$H(S^1, S^2) = \frac{1}{N} \sum_{i=1}^{N} |S_i^1 - S_i^2| \tag{I}$$

where S^1 and S^2 are the two strings rewritten by GRN architectures, $H(\cdot)$ is the Hamming distance function, and N is the length of the strings.
The ODE framework of SynTReN is given by the equation below ([1]):

$$v = \frac{V_{0,max} + \sum_{i=1}^{P} (\frac{A_i}{K_i})^{n_i^{act}} \cdot \prod_{j=1}^{P \neq i} (1 + (\frac{A_j}{K_j})^{n_i^{act}}) \cdot V_{i,max}}{\prod_{i=1}^{P} (1 + (\frac{A_i}{K_i})^{n_i^{act}}) \cdot \prod_{j=1}^{Q} (1 + (\frac{I_j}{K_j})^{n_j^{inh}})}. \tag{J}$$

where $V_{0,max}$ is the leakage transcription rate, $V_{i,max}$ is the maximal transcription rate for each possible promoter state, n_i^{act} and n_j^{inh} are the Hill coefficients for activators and repressors, K_i and K_j are the dissociation constants, A_i is the activator concentration while I_j is the repressor concentration.

$$I = I \cdot (1 + PerturbationPower \cdot U(-1, 1)). \tag{K}$$

The consensus network of 30 inferences was calculated using the following equation:

$$\overrightarrow{AM_{consensus}} = \frac{\sum_{i=1}^{30} \overrightarrow{AM_i} \cdot \frac{1}{AttractorDistance_i}}{\sum_{i=1}^{30} \frac{1}{AttractorDistance_i}}, \tag{L}$$

where $\overrightarrow{AM_{consensus}}$ is the adjacency matrix of the consensus network, $\overrightarrow{AM_i}$ is the ith outcome network, and the $\frac{1}{AttractorDistance_i}$ represents the fitness of the ith outcome network.

Table A. Kinetic parameters used for *in silico* and real-life tests

Kinetic parameter	Derived values	Reference
mRNA elongation rate	4.8 nt./s	[2]
Ribosome elongation rate	8 aa./s	[3]
mRNA degradation rate	0.0067/s	[4]
Protein degradation rate	0.00796/s	[5]

Table B. *C. albicans* strains used in this study

Description	AHY	TF	Genotype	Refe
a/Δ *wildtype*	304	WT	*a*/Δ*MTLalpha::ARG4* *C.m.LEU2*/Δ*leu2*<br>*C.d.HIS1*/Δ*his1* *URA3/ura3D::*imm^{434}<br>*IRO1/iro1*Δ*::*imm^{434} *arg4::hisG/arg4::hisG*	[6]
Δ/Δ*wor1*	856	Wor1	*a*/Δ*MTLalpha::ARG4* *C.m.LEU2*/Δ*leu2*<br>*C.d.HIS1*/Δ*his1* *URA3/ura3D::*imm^{434}<br>*IRO1/iro1*Δ*::*imm^{434} *arg4::hisG/arg4::hisG*<br>Δ*orf19.4884(wor1)::C.a.HIS1*/Δ*orf19.4884(wor1)::C.a.LEU*	[6]
Δ/Δ*wor2*	736	Wor2	*a*/Δ*MTLalpha::ARG4* *C.m.LEU2*/Δ*leu2*<br>*C.d.HIS1*/Δ*his1* *URA3/ura3D::*imm^{434}<br>*IRO1/iro1*Δ*::*imm^{434} *arg4::hisG/arg4::hisG*<br>Δ*orf19.5992(wor2)::C.a.HIS1*/Δ*orf19.5992(wor2)::C.a.LEU*	[6]
Δ/Δ*wor3*	850	Wor3	*a*/Δ*MTLalpha::ARG4* *C.m.LEU2*/Δ*leu2*<br>*C.d.HIS1*/Δ*his1* *URA3/ura3D::*imm^{434}<br>*IRO1/iro1*Δ*::*imm^{434} *arg4::hisG/arg4::hisG*<br>Δ*orf19.467(wor3)::C.a.HIS1*/Δ*orf19.467(wor3)::C.a.LEU2*	[6]
Δ/Δ*wor4*	861	Wor4	*a*/Δ*MTLalpha::ARG4* *C.m.LEU2*/Δ*leu2*<br>*C.d.HIS1*/Δ*his1* *URA3/ura3D::*imm^{434}<br>*IRO1/iro1*Δ*::*imm^{434} *arg4::hisG/arg4::hisG*<br>Δ*orf19.6713(wor4)::C.a.HIS1*/Δ*orf19.6713(wor4)::C.a.LEU*	[6]
Δ/Δ*efg1*	836	Efg1	*a*/Δ*MTLalpha::ARG4* *C.m.LEU2*/Δ*leu2*<br>*C.d.HIS1*/Δ*his1* *URA3/ura3D::*imm^{434}<br>*IRO1/iro1*Δ*::*imm^{434} *arg4::hisG/arg4::hisG*<br>Δ*orf19.610(efg1)::C.a.HIS1*/Δ*orf19.610(efg1)::C.a.LEU2*	[6]
Δ/Δ*ahr1*	812	Ahr1	*a*/Δ*MTLalpha::ARG4* *C.m.LEU2*/Δ*leu2*<br>*C.d.HIS1*/Δ*his1* *URA3/ura3D::*imm^{434}<br>*IRO1/iro1*Δ*::*imm^{434} *arg4::hisG/arg4::hisG*<br>Δ*orf19.7381(ahr1)::C.a.HIS1*/Δ*orf19.7381(ahr1)::C.a.LEU2*	[6]
Δ/Δ*czf1*	784	Czf1	*a*/Δ*MTLalpha::ARG4* *C.m.LEU2*/Δ*leu2*<br>*C.d.HIS1*/Δ*his1* *URA3/ura3D::*imm^{434}<br>*IRO1/iro1*Δ*::*imm^{434} *arg4::hisG/arg4::hisG*<br>Δ*orf19.3127(czf1)::C.a.HIS1*/Δ*orf19.3127(czf1)::C.a.LEU2*	[6]
Δ/Δ*ssn6*	801	Ssn6	*a*/Δ*MTLalpha::ARG4* *C.m.LEU2*/Δ*leu2*<br>*C.d.HIS1*/Δ*his1* *URA3/ura3D::*imm^{434}<br>*IRO1/iro1*Δ*::*imm^{434} *arg4::hisG/arg4::hisG*<br>Δ*orf19.6798(ssn6)::C.a.HIS1*/Δ*orf19.6798(ssn6)::C.a.LEU2*	[6]
Δ/Δ*rbf1*	793	Rbf1	*a*/Δ*MTLalpha::ARG4* *C.m.LEU2*/Δ*leu2*<br>*C.d.HIS1*/Δ*his1* *URA3/ura3D::*imm^{434}<br>*IRO1/iro1*Δ*::*imm^{434} *arg4::hisG/arg4::hisG*<br>Δ*orf19.5558(rbf1)::C.a.HIS1*/Δ*orf19.5558(rbf1)::C.a.LEU2*	[6]

Δ/Δ*wor1* Δ/Δ*ssn6*	1355	Wor1 Ssn6	*a*/Δ*alpha* *C.m.LEU2*/Δ*leu2* *C.d.HIS1/his1*Δ *URA3/ura3D::imm*^{434} *IRO1/iro1*Δ*::imm*^{434} *arg4::hisG/arg4::hisG* Δ*MTLalpha::ARG4* Δ*orf19.6798(ssn6)::C.a.HIS1*/Δ*orf19.6798(ssn6)::C.a.LEU2* Δ*wor1*/Δ*wor1*
Δ/Δ*wor1* Δ/Δ*rbf1*	1354	Wor1 Rbf1	*a*/Δ*alpha* *C.m.LEU2/leu2*Δ *C.d.HIS1/his1*Δ *URA3/ura3D::imm*^{434} *IRO1/iro1*Δ*::imm*^{434} *arg4::hisG/arg4::hisG* Δ*MTLalpha::ARG4* Δ*orf19.5558(rbf1)::C.a.HIS1*/Δ*orf19.5558(rbf1)::C.a.LEU2* Δ*wor1*/Δ*wor1*

Table C. Probabilities of cumulative attractor distance by the null model

	Attractor distances				
Number of genes	≤ 0.1	≤ 0.16	≤ 0.2	≤ 0.3	≤ 0.4
5 genes	0.61%	5.03%	12.50%	45.10%	73.39%
6 genes	0.29 %	3.61 %	10.53%	44.77%	74.11%
7 genes	0.14 %	2.63 %	9.00%	44.55 %	74.68%
8 genes	0.07%	1.95%	7.77%	44.36%	75.08%
9 genes	0.03%	1.46%	6.75%	44.23%	75.42%

Table C shows the probabilities of cumulative attractor distances produced by a null model. For each gene, the null model randomly picks a value in a continuous uniform distribution $\mathbf{U}([R]_{i,min}, [R]_{i,max})$, where $[R]_{i,min}$ and $[R]_{i,max}$ are the minimal and maximal expression levels of the ith gene.

Table D. Comparison of inference software features

Ability to infer	ARACNE	CLR	MRNET	MRNETB	SIMONE	GENIE3	EA
Directed GRN	No	No	No	No	No	Yes	Yes
Sign of regulation	No	No	No	No	No	No	Yes
Self-regulation	No	No	No	No	No	No	Yes
Characterization of protein-protein coordination	No	No	No	No	No	No	Yes

Table E. The *in silico* test result for protein coordination matrix

in silico GRN instances	Hamming distance	Percentile	Accuracy	Precision	Recall
5-gene GRN	1.00(5.00)	1.08%(62.37%)	0.90(0.50)	1.00(0.82)	0.90(0.50)
6-gene GRN	3.00(6.00)	7.30 %(61.29%)	0.75(0.50)	1.00(1.00)	0.75(0.50)
7-gene GRN	3.00(7.00)	2.87%(60.47%)	0.79(0.50)	0.73(0.76)	0.79(0.50)
8-gene GRN	1.5(8.00)	0.12%(59.82%)	0.88(0.50)	0.88(0.88)	0.88(0.50)
9-gene GRN	4.00(9.00)	1.54%(59.27%)	0.78(0.50)	0.60(0.65)	0.78(0.50)

Values in parenthesis show the results of random LG. Accuracy, precision, and recall are calculated by weighted average (averaging the support-weighted mean per label).

Table F. The *in silico* test result for $f0$

in silico GRN instances	Average $f0$	Std $f0$
5-gene GRN	[0.001, 0.016, 0.013, 0.042, 0.001]	8.28e-2
6-gene GRN	[0.023, 0.043, 0.018, 0.07, 0.004, 0.028]	1.42e-1
7-gene GRN	[0.039, 0.045, 0.046, 0.052, 0.045, 0.015, 0.019]	1.28e-1
8-gene GRN	[0.028, 0.029, 0.031, 0.228, 0.021, 0.036, 0.074, 0.3]	1.84e-1
9-gene GRN	[0.006, 0.021, 0.031, 0.27, 0.098, 0.054, 0.086, 0.095, 0.035]	1.14e-1

The f_0s are 0 for all *in silico* reference GRNs.

Table G. Variance of the outcome networks in the *in silico* test

Network size	5 genes	6 genes	7 genes	8 genes	9 genes
Variance	0.214	0.275	0.327	0.335	0.265

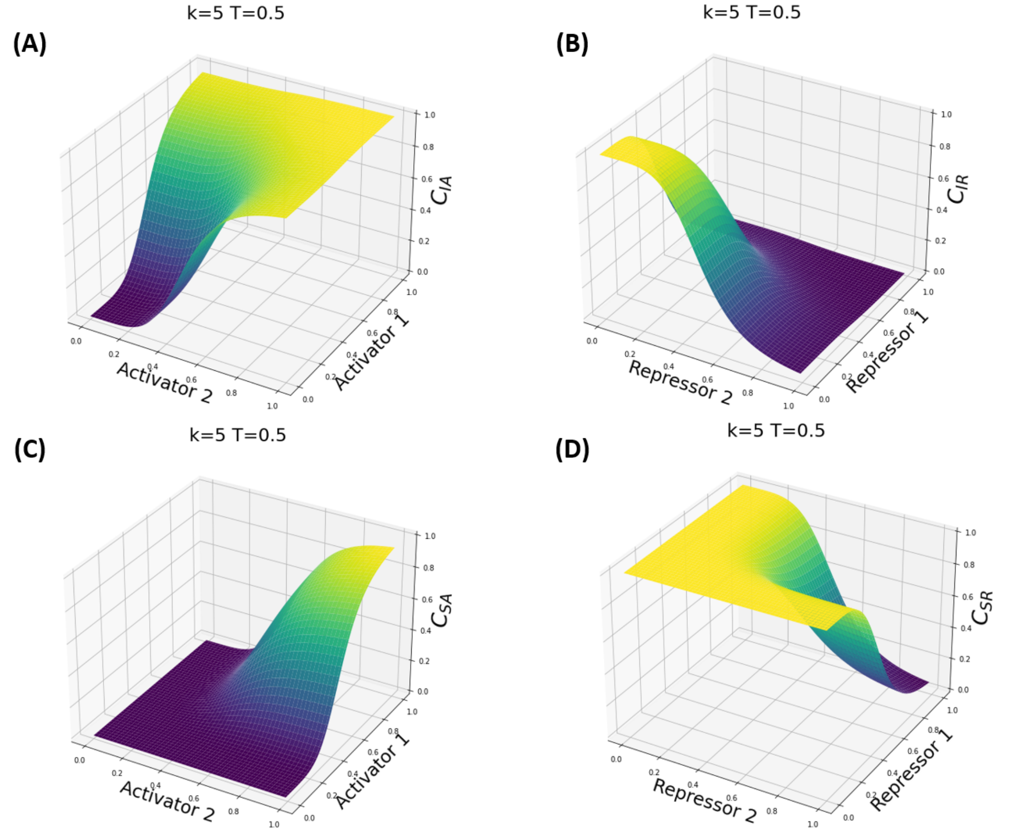

Fig A. Demonstration of multiple-TF regulation. The x-axis and y-axis are the protein concentration of two activators or repressors, and the z-axis shows the outcome of the regulation function. (A) Two independent activators. (B) Two independent repressors. (C) Two synergistic activators. (D) Two synergistic repressors.

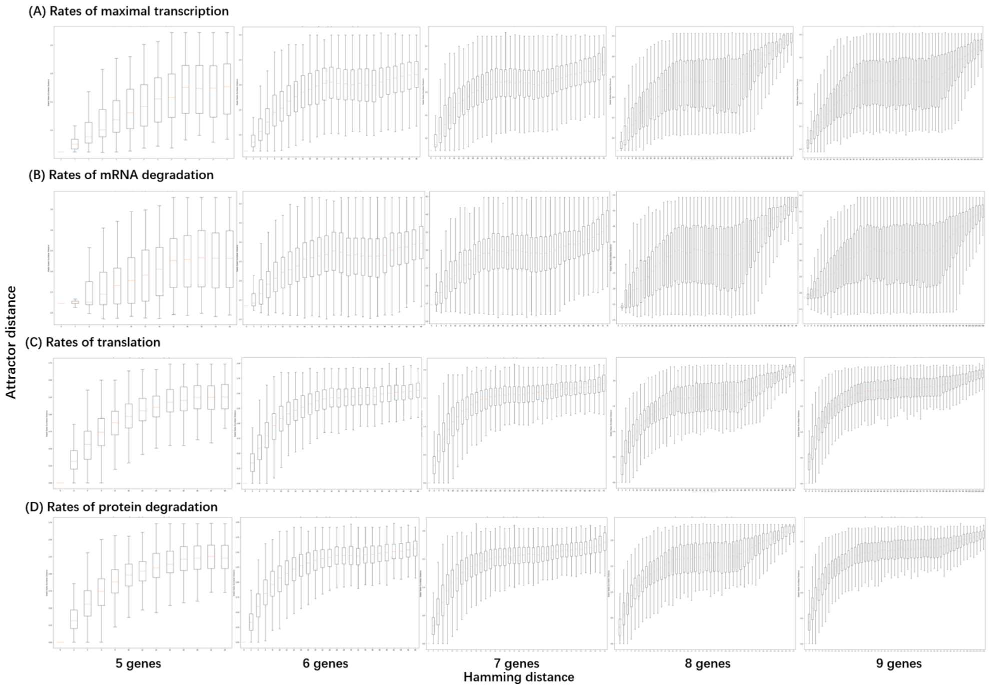

Fig B. Sensitivity tests for the kinetic parameters, (A) rates of transcription, (B) mRNA degradation, (C) translation, and (D) protein degradation. Each of these parameters was perturbed by 50% of their original values and used to generate the correlation between A_{net} similarity (Hamming distance on the horizontal axis) and attractor profiles similarity (attractor distance on the vertical axis).

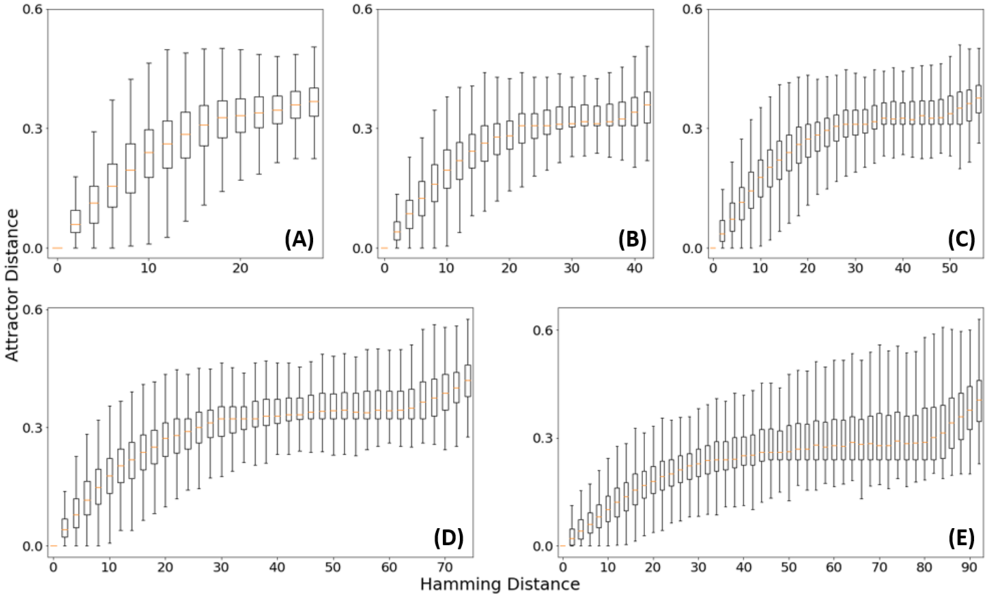

Fig C. Positive correlation between A_{net} similarity (Hamming distance on the horizontal axis) and attractor profiles similarity (attractor distance on the vertical axis). Each column in the box plots (A-E) contains 1000 random A_{net}^{mut} mutated from the 5 A_{net}^{ref} consisting of 5-9 genes.

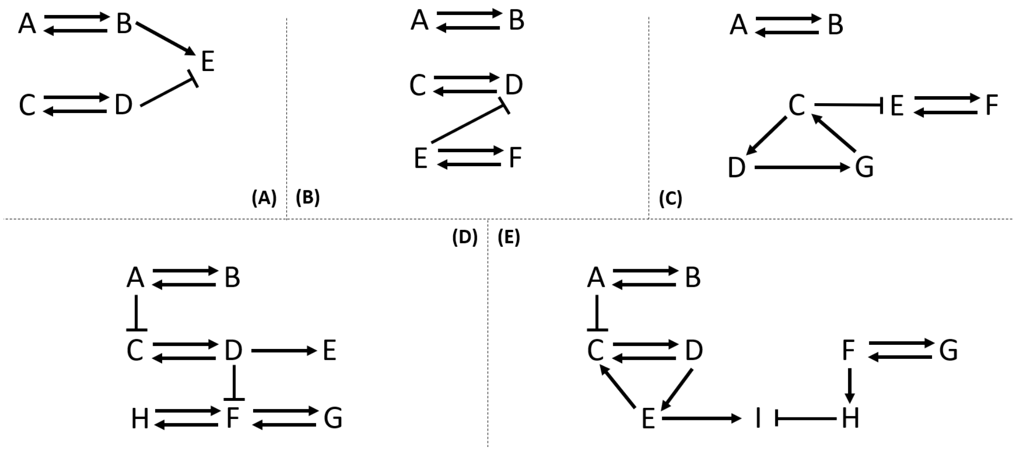

Fig D. Five GRN architectures were arbitrarily generated as references in the *in silico* test. They have five-nine (A-E) genes and no self-regulatory edges. The pointed arrows represent activating and the blunt arrows represent repressing regulatory interactions.

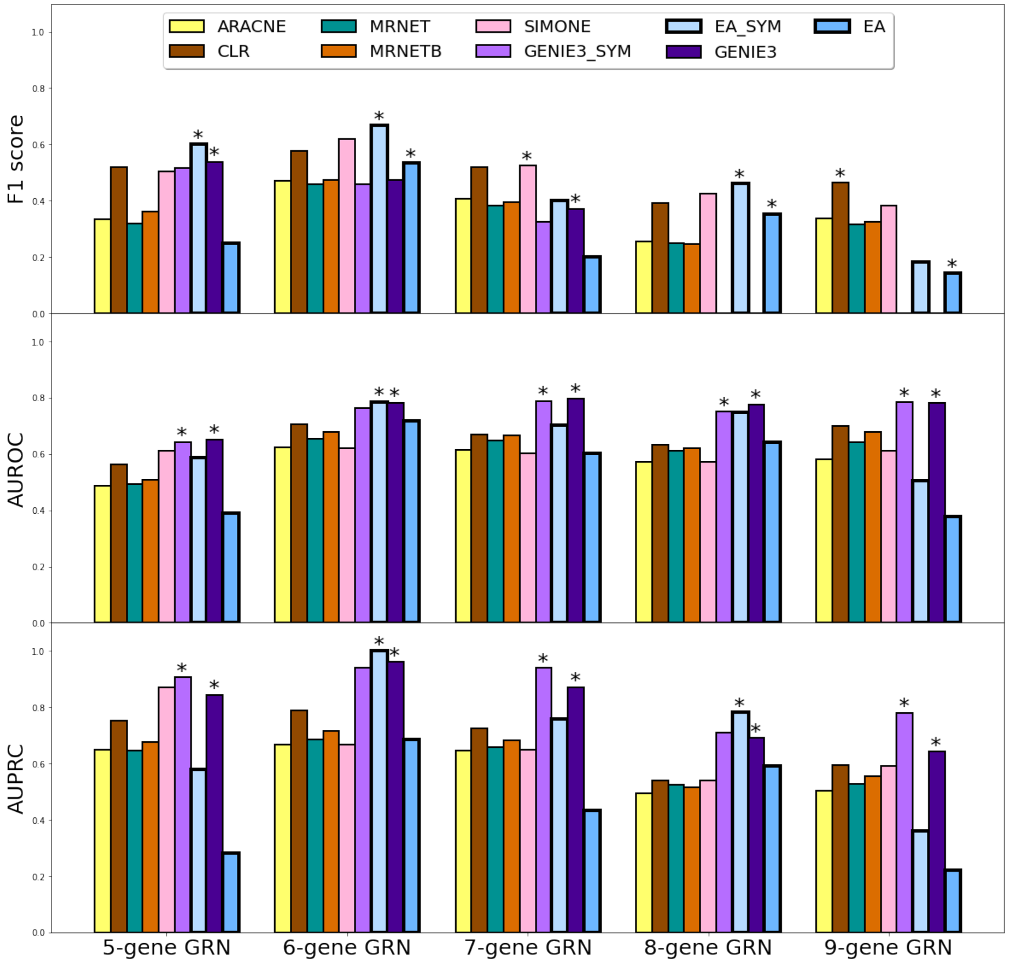

Fig E. The non-autoregulation *in silico* test comparison results in F1 score (upper panel), AUROC (middle panel), and AUPRC (bottom panel). The F1 scores are calculated using a threshold cutoff of 0.5 for all models. Best performances are marked by asterisks for symmetric and asymmetric methods.

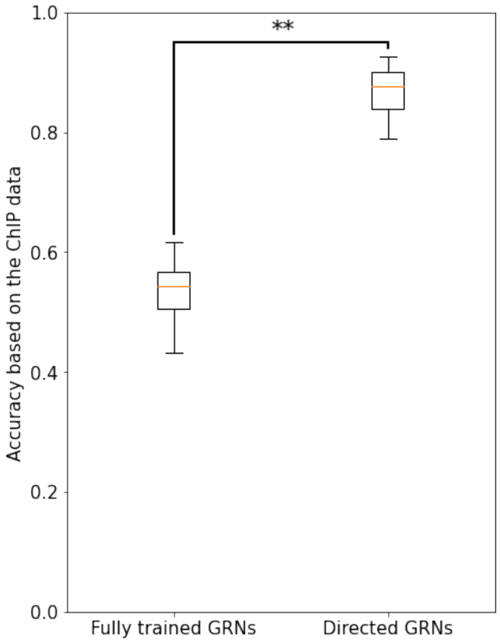

Fig F. Accuracy distributions of the fully trained and directed GRNs determined by the ChIP data in *C. albicans*. Each distribution contains 30 GRN samples. The fully trained GRNs were solely inferred by the transcriptional profiles while the directed GRNs were also constrained by the ChIP data. Performing equally well on reproducing the transcriptional profiles, the direct GRNs showed a significant increase compared to the fully trained GRNs.

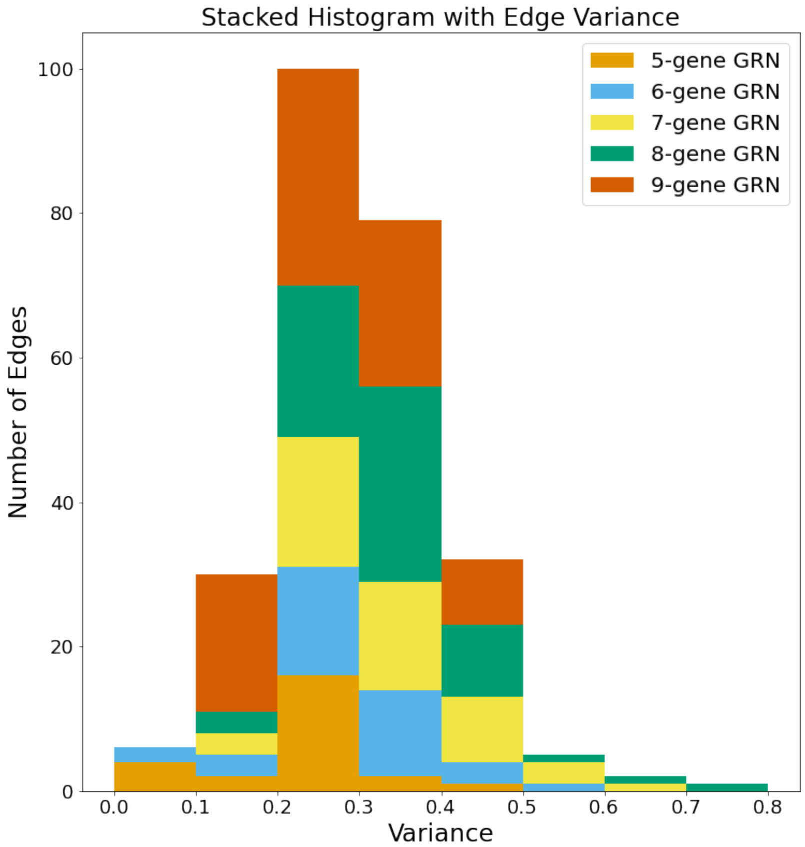

Fig G. A stacked histogram displays the distribution of edge variances across 30 independent inference runs, showing the number of edges for each variance category.

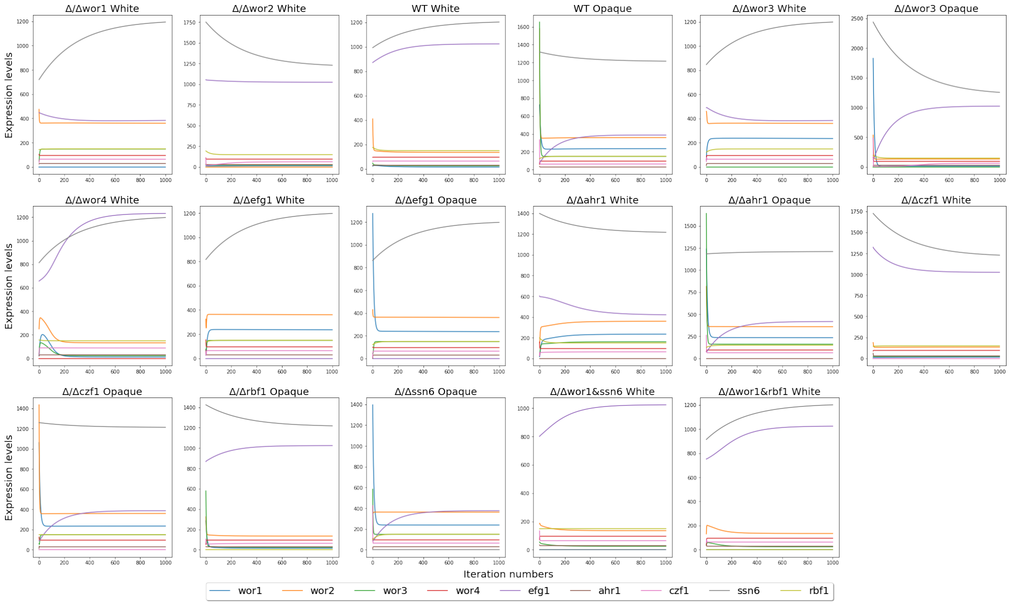

Fig H. Prediction of drop-out transcriptional profiles in *C. albicans*. A dropout strategy was utilized to infer GRNs based on a subset of available data and assessed the predictive capability of the inferred GRNs for transcriptional profiles that were deliberately excluded from the training dataset. The initial states were configured to correspond to the omitted transcriptional profiles.

References

1. Van den Bulcke T, Van Leemput K, Naudts B, van Remortel P, Ma H, Verschoren A, et al. SynTReN: a generator of synthetic gene expression data for design and analysis of structure learning algorithms. BMC bioinformatics. 2006;7(1):43.

2. Klumpp S, Hwa T. Growth-rate-dependent partitioning of RNA polymerases in bacteria. Proceedings of the National Academy of Sciences. 2008;105(51):20245–20250.

3. Guet CC, Bruneaux L, Min TL, Siegal-Gaskins D, Figueroa I, Emonet T, et al. Minimally invasive determination of mRNA concentration in single living bacteria. Nucleic acids research. 2008;36(12):e73–e73.

4. Moran MA, Satinsky B, Gifford SM, Luo H, Rivers A, Chan LK, et al. Sizing up metatranscriptomics. The ISME journal. 2013;7(2):237–243.

5. Maurizi M. Proteases and protein degradation inEscherichia coli. Experientia. 1992;48(2):178–201.

6. Lohse MB, Ene IV, Craik VB, Hernday AD, Mancera E, Morschhäuser J, et al. Systematic genetic screen for transcriptional regulators of the Candida albicans white-opaque switch. Genetics. 2016;203(4):1679–1692.
